# Unveiling the noxious effect of polystyrene microplastics in aquatic ecosystems and their toxicological behavior on fishes and microalgae

**DOI:** 10.3389/ftox.2023.1135081

**Published:** 2023-05-04

**Authors:** Nurin Nabilah Jalaudin Basha, Nurfarwizah Binti Adzuan Hafiz, Mohamed Syazwan Osman, Noor Fitrah Abu Bakar

**Affiliations:** ^1^ EMZI-UiTM Nanoparticles Colloids & Interface Industrial Research Laboratory (NANO-CORE), Chemical Engineering Studies, College of Engineering, Universiti Teknologi MARA, Cawangan Pulau Pinang, Permatang Pauh Campus, Pulau Pinang, Malaysia; ^2^ School of Chemical Engineering, College of Engineering, Universiti Teknologi MARA Shah Alam, Shah Alam, Malaysia

**Keywords:** polystyrene microplastics, abundance, identification, toxicological, microalgae, fishes

## Abstract

Microplastic (MP) particles are considered noxious pollutants due to their presence in aquatic habitats at almost every level of the food chain. Thus, the entry of MP particles into marine waterbodies has triggered a common research interest. Until recently, the toxicity of polystyrene towards aquatic creatures in comparison to other polymers has not been widely investigated. This article provides an extensive overview of the occurrence of microplastic particles, the route of polystyrene (PS) in the aquatic ecosystem, the PS properties characterization, and its noxious effects on the aquatic biota, particularly fishes and microalgae. Alarming high levels of polystyrene were found in urban, coastal, and rural surface waters and sediments. The fast-screening technique began with a stereoscope to determine the polystyrene particles’ shape, size, and color on the organism. SEM and complemented by micro FTIR or Raman spectroscopy were used to evaluate MP’s polymer structures. The findings present evidence suggesting that polystyrene buildup in fish can have long-term and unknown consequences. Meanwhile, the presence of polystyrene on microalgae causes a decrease in chlorophyll concentration and photosynthetic activity, which may disrupt photosynthesis by interfering with the electron characters and leading to the production of reactive oxygen species (ROS).

## Introduction

Due to further technical and medical advancements, plastics offer a variety of advantages for society in daily life. Nevertheless, plastic consumption contributes to environmental pollution given its poor biodegradability, improper applications, and ineffective disposal (Mariano et al., 2021). The approaches of disposing of plastics results in the buildup of trash in landfills and natural habitats, thereby creating physical problems for animals that eat or get tangled in plastic, leaching of chemicals from plastic products, and the possibility of transferring chemicals to people and animals (Thompson et al., 2009). These events highlight the public health significance of proper disposal of plastic products. Guerrero and his team also identified insufficient waste management programs in most cities as the main cause of the massive amount of solid waste in the freshwater ecosystems (Guerrero et al., 2013).

Microplastics (MPs) is the general word for smaller plastic components, especially their microscopic versions that are less than 5 mm in size. Plastics that are already small, to begin with, are found in produced products such as cosmetics, detergents, drug vectors, and air-blasting media and are known as “microbeads” or primary MPs. These particles are broken down into plastic microparticles and nanoparticles by the Sun’s ultraviolet (UV) radiation and by physical forces (Gewert, Plassmann, and Macleod, 2015). There are many kinds of polymers, such as polypropylene (PP), polyethylene (PE), polystyrene (PS), polycarbonate (PC), rubber, and polyvinyl chloride (PVC). Most of the microplastics result in relatively the same impact caused by exposure to a wide variety of novel chemical pollutants over the past decades. This is the result of high consumption, lack of regulation, and inadequate waste management of commercial items, and today they are viewed as ecological and health risks (de Souza Machado et al., 2018).

Due to their greater surface-to-volume ratio, MPs are very vulnerable to absorbing organic pollutants and pathogens from their surroundings than larger plastic particles (Gopinath et al., 2020). Furthermore, plasticizers, flame retardants, antioxidants, surfactants, and thermal stabilizers adsorb organic contaminants from the surrounding environment, increasing exposure to a variety of poisons (Wagner et al., 2014). Most plastics also release estrogenic chemicals into the environment, which could have deleterious effects on human health (Yang et al., 2011). Thus, several noxious routes are required for MPs to penetrate the aquatic environment. Therefore, the rising concerns regarding MPs in freshwater ecosystems are valid and warrant more investigation.

Despite extensive MP research worldwide, there is a dearth of information on the abundance and specific toxicity of polystyrene. This study focuses on the aquatic environment, and the phrases “aquatic environment” and “aquatic ecosystem” refer to bodies of water in the United States, such as wetlands, that are home to communities and populations of interdependent or mutualistic plants and animals. The aquatic system also emphasizes the ecology of freshwater, saltwater, estuaries, and oceans (Duarte, 2013). Given the rising production and expanding consumption, the aquatic environment has turned into a barrel of trash for all types of plastic, endangering the habitat and essential functions of both inland and offshore ecosystems. This situation highlights the pollution caused by plastics on the global stage and reflects a growing global environmental issue that could have negative impacts on human health and biological diversity since plastics can be progressively broken down into microscopic fragments and considered hazardous to aquatic life (Mukhopadhyay and Valsalan, 2022). Therefore, the review aims to provide a comprehensive overview of the occurrence of MPs in the aquatic environment, the route of PS into the aquatic ecosystem, and the method of identification of PS. This article also looks into the noxious effects of PS in freshwater ecosystems, focusing on PS as the MPs model.

## Accumulation and abundance of microplastics in aquatic environment

Research into microplastics in water and sediment was conducted at Red Hills Lake in 2020 using a protocol developed by the National Oceanic and Atmospheric Administration (NOAA). This lake is one of the freshwater systems that supply water to the northern part of Chennai city. With an average concentration of 27 particles/kg in sediment and 5.9 particles/L in water, the most prevalent types of MPs were fibers (37.9%), pieces (27%), films (24%), and pellets (24%) (11.1%) (Gopinath et al., 2020). Rowley et al. (2020) found MPs in the water column samples of the River Thames in the United Kingdom. The concentration was 14.2 per m^3^ at Greenwich (downstream) and 24.8 per m^3^ at Putney (upstream), which is considered among the highest concentrations ever recorded worldwide. The most common polymers found in River Thames were polyethylene and polypropylene, which suggests broken packaging as the main source of MPs in these samples. This finding is alarming since the concentrations are analogous to the highest recorded in the world. Specifically, the samples were obtained from June to October 2017 during the ebb and flood tides at the surface and a depth of 2 m (Rowley et al., 2020).

Concurrently, Yang Liu Y et al. (2020) used the μ-FTIR method to analyze the mainstream distribution and features of MPs in Haihe River, which flows through a densely populated urban area and eventually empties into the Bohai Sea. The number of MPs per cubic meter ranged from 0.69 to 74.95, with polyethylene, poly (ethylene-propylene) copolymer, and PP accounting for the main components (Liu G et al., 2020). A study on the Surabaya River in Indonesia assessed the distribution of MPs at the surface, idle and bottom levels of the river (Lestari et al., 2020). The primary purpose of the Surabaya River is to provide a source of raw water for the city of Surabaya. In addition, this river is utilized for irrigation purposes and the outflow of waste from both homes and businesses. The MP concentration ranged from 1.47 to 43.11 for the surface samples, 0.76 to 12.56 for the middle samples, and from 1.43 to 34.63 particles/m^3^ for the bottom samples. The highest average of MPs was located at the lower end of the river. Specifically, the main polymer types of MPs discovered in the river were low-density polyethylene (LDPE), polypropylene (PP), and polyethylene (PE).

In Lake Victoria, East Africa, the distribution of MPs was examined on three sites. Group A sites were fish landing and recreational beaches, located in an urban or semi-urban setting. Group B sites were only fish landing beaches found in a rural community. Group C sites were river discharges. According to the researchers, MPs were detected in all the sites (range: 2834 ± 329,167 particles/km^2^ or 0.02 ± 2.19 particles/m^3^). MPs were found to be most abundant in group A (range: 103,333 ± 329,167 particles/km^2^ or 0.69 ± 2.19 particles/m^3^) and least abundant in group C (range: 283 ± −20,840 particles/km^2^ or 0.02 ± 0.14 particles/m^3^). MPs made of polyethylene and polypropylene, which are known as secondary MPs, were predominant (Egessa et al., 2020). The researchers posited that recreational activities performed proximal to Lake Victoria were a major factor contributing to a huge amount of MPs in the freshwater as most of the MPs were derived from plastic materials used by the nearby community. A similar study discovered that the Wei River’s low water flow and high sand content influenced the buildup of MPs in both water and sediment. The surface of the river recorded a concentration of 3.67–10.7 items per liter of MPs, whilst the sediments had 360 to 1,320 items per kilogram of MPs (Ding et al., 2019).

As mentioned in the literature review focusing on the Tuojiang River basin in southwest China, the presence of polypropylene as the main polymer contaminant was detected in all seven cities that make up the Tuojiang River basin (Zhou et al., 2020). The concentrations of these MPs ranged from 911.57 ± 199.73 to 3395.27 ± 707.22 items/m^3^, with the highest MPs concentration observed in the urban water of Ziyang. One form of typical and prevalent MP was fiber, which ranged from 34.88% to 65.85%. Among all of the samples, the most common dimension was between 0.5 and 1 mm (27.27%–66.67%), while the most common hue was white (23.30%–54.29%). Scanning electron microscope analysis (SEM) revealed that the surfaces of the MPs had numerous fissures, which absorbed a high number of particles (Zhou et al., 2020).

Extensive research by He et al. (2020) revealed that PE, polyamide (PA), and PP were the main contaminants found in Brisbane River with polyethylene terephthalate (PET) as the side polymer type. The investigation was conducted over several seasons, and it was found that dry and rainy seasons were primarily characterized by low and high concentrations of MP abundance, respectively. The abundance ranged from 10 to 520 items per kilogram, or 0.18–129.20 mg kg^−1^. MPs were found to be more prevalent in the dry season (930 items/L) than in the rainy season (497 items/L) (He et al., 2020). The same conclusion was reached by Fan et al. (2019) in which the river water had more MPs in the dry season than in the wet season, suggesting that precipitation dilutes the concentration of the microorganisms.

Fan et al. (2019) investigated the sediments of China’s Pearl River and its tributaries. Resultantly, MPs were found in abundance in all three sediment types. MP abundances were determined to be 0.57 ± 0.71 items L^−1^ in river water, 685 ± 342 items kg^−1^ dry weight (dw) in river bed sediment, and 258 ± 133 items kg^−1^ dw in estuarine sediment. The MPs in the Pearl River catchment area might have stemmed from the fragmentation of discarded plastic wastes, as these wastes are dominated by sheet, fragmental, and fiber polyethylene, polypropylene, and ethylene-propylene copolymers. PET and polyvinyl alcohol were more prevalent in coastal silt than in river water. The Pearl River and its tributaries are anticipated to emit 15,963 tonnes of MPs—a menacing figure of release.

In Tibet Plateau, the Third Pole of the world was studied by Jiang et al. (2019). The study revealed that the number of MPs found in the surface water of the river in the Tibet Plateau ranged from 483 to 967 items per cubic meter, whereas the amount found in the sediment ranged from 50 to 195 pieces per kilogram (Jiang et al., 2019). Meanwhile, another study that investigated the amount and distribution of primary and secondary MPs in an urban river near the coastal environment in the West of Scotland found that 88% of the overall counts comprised the major fiber type, which ranged from 161 to 432 MPs kg^−1^ dry sediment (Blair et al., 2019). However, the presence of fibers in the blanks suggests that air contamination may have played a role. Fibers accumulated primarily in fractions smaller than 0.09 mm, indicating that drivers of fine sediment movements in the river may affect their fate.

Lastly, a recent study attempted to identify the occurrence, characteristic polymer type, and composition of the contaminant in Lutong Beach, Sarawak, Malaysia (Anak Alexander Tampang and Mohan Viswanathan, 2022). Lutong Beach, a popular hangout for locals and tourists alike, is particularly dense with MPs such as PS and styrene (PS), as well polyethylene (PE), polyester, and polypropylene (PP). When compared to other elements such as calcium, aluminum, titanium, and chlorine, carbon, and oxygen were found to be the most prevalent. As an additive, the most common usage of these elements is in the manufacturing process, where they are employed to improve plastic quality. To date, the debate has long prevailed due to micro-sized plastic accumulation, thus highlighting the urgent need for the characterization techniques of MPs and reporting data on their occurrence worldwide.

## Polystyrene as the main focus

Among the commonly used plastic polymers, PS has received the least amount of toxicity research. Styrene is an aromatic hydrocarbon that has a flowery odor and appears as a colorless or yellowish viscous liquid (PubCHEM, 2004). The auto-ignition temperature is 490°C, and its flash point is 34°C (in a closed cup). Its lower explosive limit is between 0.9% and 1.1% volume/volume (v/v), whereas its upper explosive limit is between 6.1% and 6.8% v/v. PS can quickly ignite when exposed to heat, sparks, or flames, and its vapors can produce explosive air mixes due to the formation of peroxides. However, degradation techniques are therefore sought after. Styrene is only considered for reuse in its original form or for burning, which requires temperatures of up to 1,000°C. Despite growing global manufacturing, studies of the accumulation of plastic in the Atlantic Ocean over the past 22 years have not discovered any increase in the amount of this material (Law et al., 2010). Styrene may polymerize when polluted with oxidizing chemicals or halides, or when heated; it releases pungent smells after being broken down. It is often stabilized for safe storage, transit, and usage using p-tert-butyl catechol (PubCHEM, 2004). In addition to ethylbenzene, common impurities include polymer content, aldehydes, peroxides (as H_2_O_2_), benzene, sulfur, and chlorides.

PS accounts for 50% of all industrial resins made from styrene, followed by styrene-butadiene rubber (15%), unsaturated polyester resins (glass-reinforced) (12%), styrene-butadiene latexes (11%), acrylonitrile-butadiene-styrene (10%), and styrene. These resins can all be placed into one of six major groups (1%). Unsaturated polyester resins (non-reinforced) are a minor type of application (Mukhopadhyay and Valsalan, 2022). PS, which is a result of the polymerization of styrene monomers, is one of the polymers that is frequently utilized in the manufacturing of Styrofoam and other goods such as toys, CD cases, and cup covers. Both *in vivo* and *in vitro* studies have led researchers to conclude that polystyrene-nanoparticles (PS-NPs) may enter organisms through a variety of pathways, including the skin, the respiratory system, and the digestive system. They can leave their mark on living organisms and build up in higher levels of the food chain (Kik et al., 2020). By using a laser with an excitation and emission wavelength of 400 and 450 nm, it was possible to create blue fluorescence in PS with a size of 200 nm (Huang et al., 2022).

## The ingestion of polystyrene by aquatic biota routes

A variety of sources are suggested to contribute to the presence of the MPs found within the aquatic samples. However, the exact sources of the MPs are impossible to identify. According to Steensgaard et al. (2017), PS plastic debris frequently settles and gathers in urban streams and landfills. Although styrene plastics are intended to be recycled by some ethical individuals, only a tiny portion of the waste is finally used. According to the statistics on municipal solid waste released by the Environmental Protection Agency in 2005, the total amount of solid PS garbage recycled in the United States reached 2.6 million tonnes. Since PS is not biodegradable, the report reflects that styrene is a big problem and a big cause of pollution in the environment. (Tokiwa et al., 2009).

There are many pathways for MPs to enter freshwater ecosystems (rivers, lakes, and streams), including sewage and landfill effluents, urban runoff, air deposition, wastewater treatment plants, and inappropriate trash disposal. Presently, rivers are the most common route for plastic waste to get into the ocean (Auta et al., 2017). Climate conditions and hydrodynamic processes in rivers, lakes, and streams, such as current, waves, wind, river discharge rate, and position, determine where MP particles are discovered and how rapidly they sink or float back to the surface in freshwater environments (Rodrigues et al., 2018). Meanwhile, in aquatic ecosystems, MPs degrade into nano plastics when subjected to UV radiation, tidal forces, and chemically or biologically-driven degradation mechanisms, leading to detrimental consequences for freshwater and marine food webs. When MPs find their way into aquatic environments, they have the potential to be absorbed by zooplankton, planktivorous, and piscivorous fish in the aquatic food web. After being transferred to the food chain, humans eventually digest them. The consumption of contaminated water, sea salts, and shellfish by humans is the primary source of particle plastics that are ingested by humans (Benson et al., 2022).

Gopinath et al. (2020) stated that the water treated from Red Hills Lake, Chennai City in India does not undergo the removal of the MP process being delivered to the citizens of Chennai. Therefore, residents may be consuming contaminated water containing MPs, eventually facilitating the channel of MPs into the aquatic environment after being digested, thereby exposing the individual to severe health hazards (Gopinath et al., 2020). In Wei River, China, researchers detected the presence of PS but the reason underlying reason remains unknown (Ding et al., 2019). The samples of the water surface and sediment in the Tiber Plateau were obtained using a big flow sampler and a shovel made of stainless steel. This contamination also occurred not only in developed areas where there is an elevated level of human activity but also in remote areas.

The reason for the relatively higher number of MPs in remote compared to urban areas is not fully understood. Accumulated evidence from research conducted in Okinawa, Japan revealed that Polychlorinated biphenyls (PCBs), dichlorodiphenyltrichloroethane (DDTs), and chlordane compounds (CHLs) were greater concentrations in oysters from southwestern populated regions of the study location. Surprisingly, in Okinawa’s northern rural region with less human and industrial activity, α-hexabromocyclododecanes (HBCDs) were found at a similar level as the urban region although oyster concentrations of PBDEs were significantly lower. Notably, the highest log bioaccumulation factors (BAF) values were discovered for HBCD, despite its lower log Kow than those of a-HBCD and PCB congeners. This was the case despite the log biota (oyster)-sediment accumulation factors (BSAF) values for HBCDs being relatively lower. Oysters in the Okinawa coastal region may be regularly exposed to micronized polystyrene foam particles containing HBCD because a-HBCD was discovered to be the main diastereomer in a few samples of polystyrene foam (Mukai et al., 2020).

Thus, evidence from the research by Mukai et al. (2020) suggests the contamination of MP in the rural area of the Tiber Plateau. Even in rural or urban areas, Tibet Plateau had undeniably been tainted with MPs (Jiang et al., 2019). Subsequently, a few studies confirmed that MPs were coming from developed areas with intense human activities and remote areas (Blair et al., 2019; Anak Alexander Tampang and Mohan Viswanathan, 2022).

## Method of evaluating the presence of polystyrene

Detection of plastic debris; PS MP (5 mm) with aquatic organisms from all trophic levels is one of the vital issues that need to be reviewed. These MPs are common in aquatic systems such as freshwater and are known to interact with aquatic organisms. Different methods have been proposed to analyze the presence of PS, which can be categorized as microscopy and analytical method (Mariano et al., 2021). In this review, we elaborate more on stereoscopy and scanning electron morphology (SEM) as the microscopy methods while FT-IR spectroscopy and Raman spectroscopy were discussed under analytical methods.

### Microscopy method

#### Stereoscopy

The stereo microscope provides a three-dimensional study by obtaining two images from slightly different perspectives (Mariano et al., 2021). This is the first step of the fast-screening method that allows for the immediate identification of the physical properties of MP such as the particles’ form, size, and color, which will be further defined by other methods (Song et al., 2015). This microscopy method can be employed to examine objects, particularly through reflected light at low magnification, 8 to 50 times. Unlike optical microscopes, stereoscopic microscopes illuminate from above while some designs include dual illumination. Overall, stereoscopic microscopes can be used to examine full fresh microorganisms, cells, fungi, and permanent slides, unlike the optical microscope with higher magnifications.

**FIGURE 1:**
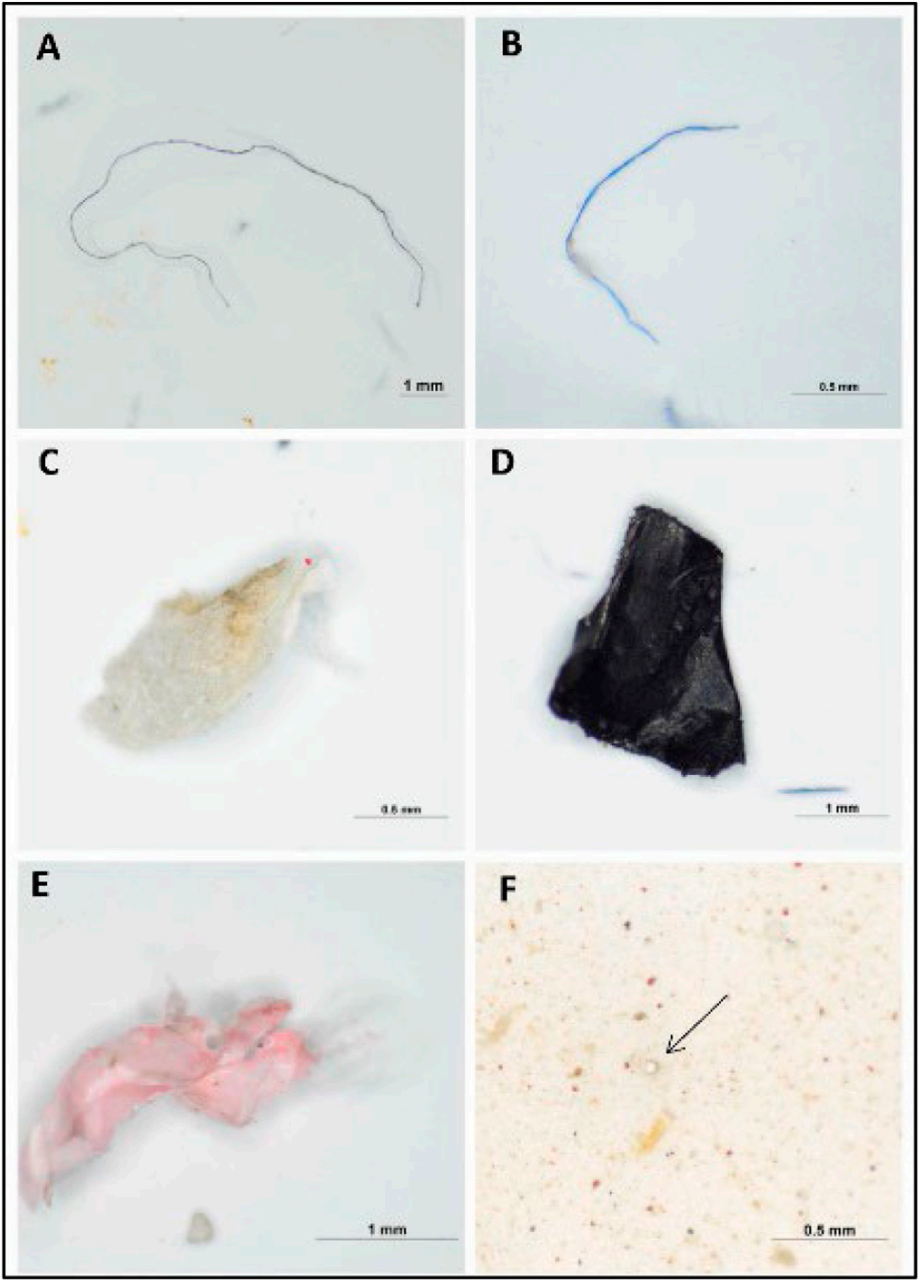
Images of forms of MPs present in the beach sediments: **(A,B)** fibers, **(C)** foam, **(D,E)** fragments, **(F)** pellet by stereo microscope (anak Alexander Tampang and Mohan Viswanathan, 2022). Reprinted from Occurrence, distribution and sources of microplastics in beach sediments of Miri coast, NW Borneo, Aliza Marai anak Alexander Tampang, Prasanna Mohan Viswanathan, (2022), with permission from Elsevier.

**FIGURE 2:**
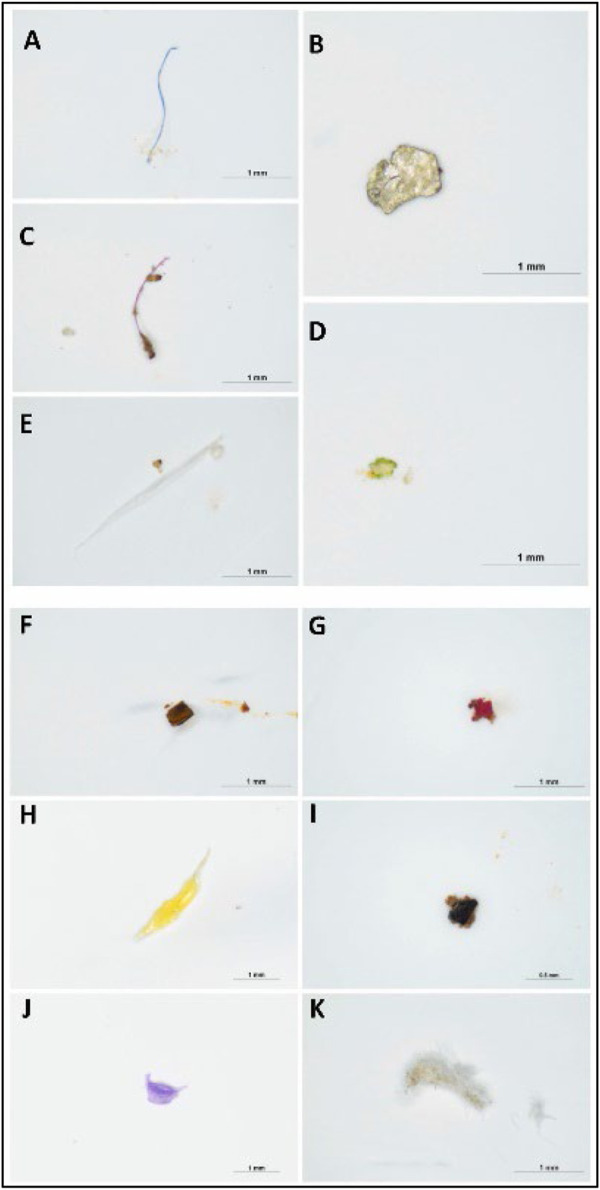
Images of different colors of MPs present in the beach sediments: **(A)** blue **(B)** white **(C)** red **(D)** green **(E)** transparent **(F)** brown **(G)** pink **(H)** yellow **(I)** black **(J)** purple **(K)** gray (anak Alexander Tampang and Mohan Viswanathan, 2022). Reprinted from Occurrence, distribution and sources of microplastics in beach sediments of Miri coast, NW Borneo, Aliza Marai anak Alexander Tampang, Prasanna Mohan Viswanathan, (2022), with permission from Elsevier.

Anak Alexander Tampang and Mohan Viswanathan (2022) employed stereoscopic microscopes to identify the abundance of MP in Miri. Briefly, the sediments were dried in the laboratory oven at a temperature of 70°C for one night as part of the pre-extraction process. After the samples were dried and sieved, 30 g of the sediment samples were obtained using a cone and quartering technique for the extraction of MPs. The separated sediment samples were placed in a beaker containing 400 mL of hydrogen peroxide (H_2_O_2_) and allowed to soak for an extended period to eliminate any organic debris. After the sediment had settled, the density separation procedure was utilized once more, this time with zinc chloride as the medium, to separate the MPs from the samples. Thereafter, the sediment samples were filtered and the filter paper was placed in a petri dish and allowed to air-dry before the next step. In the next stage, the stereomicroscopic quantification of MPs and the study of their physical features were performed. The SMZ745T Nikon Stereo Microscope was outfitted with a camera and connected to a computer to enable early observations regarding the different sorts of MPs and their forms (Figure 1) and colors (Figure 2).

Although the stereomicroscope’s lower magnification may seem like a limitation, it is advantageous in terms of simplicity for investigating samples with unaided eyes. A stereoscopic microscope is an important tool that is utilized frequently in identifying MPs whose diameters lie within the region of hundreds of microns. Even though numerous particles with a size of a few microns are visible under a microscope, it is difficult to define clear or shaped particles smaller than 100 m. In addition, extremely dense silt samples can hinder the identification of MPs on a filter paper using a microscope If a sample contains substances that cannot be removed through chemical digestion, the identification is jeopardized.

Several studies demonstrated that for transparent particles, the proportion of plastic-like particles detected by stereomicroscope and subsequently explained by other methodologies ranged from 20% to 70% of the total particles observed. A stereo microscope is unable to distinguish between synthetic and natural fibers, which are abundant in water, sediment, and biota samples. MPs are identified using a stereomicroscope based on their physical appearances. Nevertheless, it is necessary to combine stereo microscopy with other techniques such as spectroscopy (Mariano et al., 2021; Song et al., 2015).

#### Scanning electron morphology (SEM)

Researchers have also used SEM to gain more insight into the morphological surface structure of MPs and generate high-resolution photographs of the surface condition of the material. SEM uses an electron probe with an energy of up to 40-kilo electron volts (keV) to concentrate on a sample and scan it in a grid pattern of parallel lines. The impact of the incident electrons generates a variety of signals, which are then gathered to create an image or analyze the sample surface. High-energy backscattered electrons from the primary beam are the most common secondary electrons, with energies in the tens of eV or less. Additionally, it can provide information on the samples’ chemical composition with the use of EDS (Energy Dispersive X-ray Spectroscopy) detectors (Woo et al., 2021).

**FIGURE 3:**
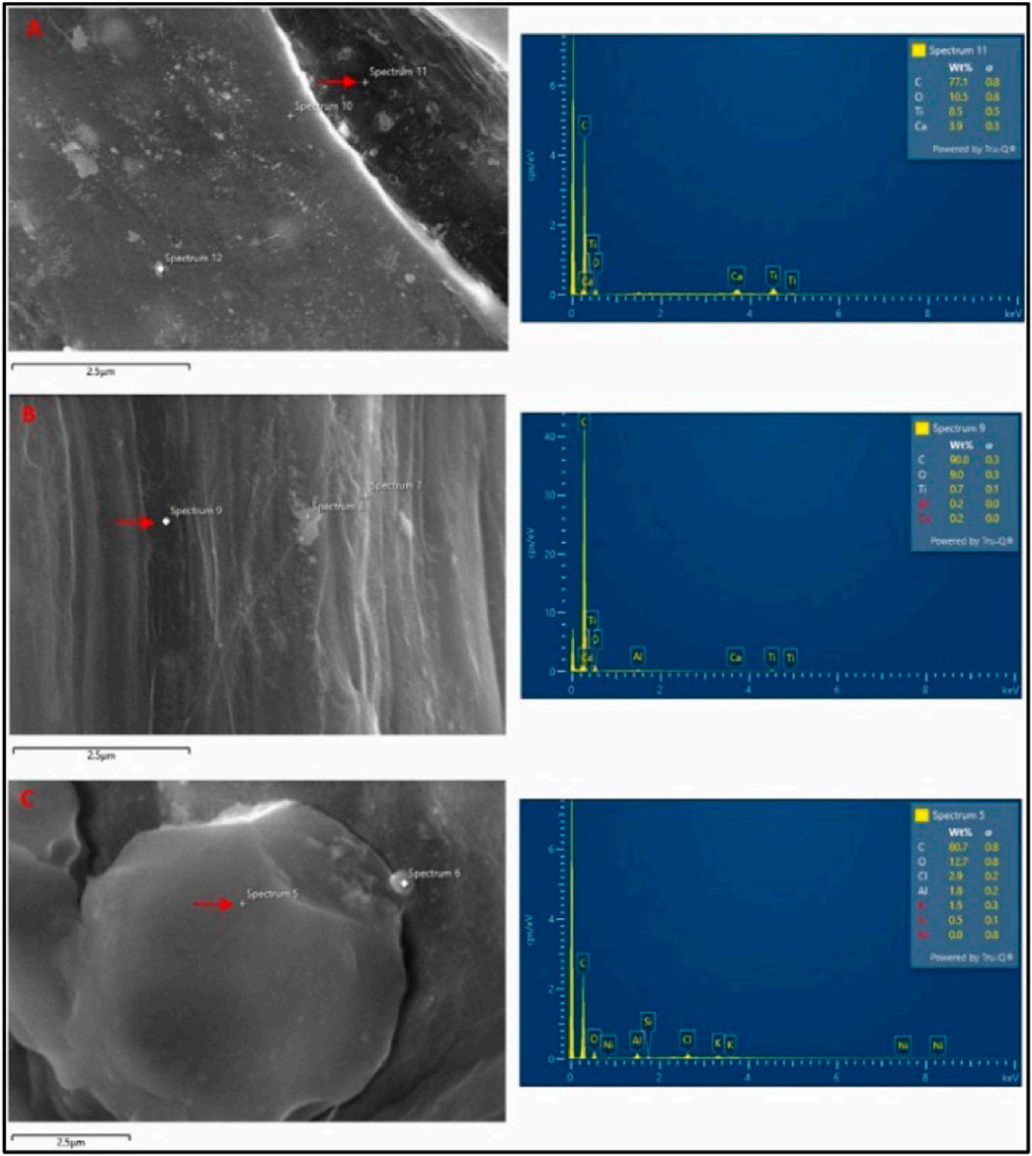
SEM image and EDX spectra of **(A)** Fragment, **(B)** Fiber and **(C)** Foam. Reprinted from Occurrence, distribution, and sources of microplastics in beach sediments of Miri coast, NW Borneo, Aliza Marai anak Alexander Tampang, Prasanna Mohan Viswanathan, (2022), with permission from Elsevier.

Research by Anak Alexander Tampang and Mohan Viswanathan (2022) entailed the use of SEM to evaluate the distribution of MPs in Miri Beach sediments, NW Borneo. The microphotographs of the extracted MPs were taken with an SEM equipped with an Energy Dispersive X-Ray (EDX) to investigate the elemental composition of the MPs. Furthermore, images obtained using an SEM displayed the rough and irregular surfaces of the fragmented MPs. This has been shown in Figure 3 below.

The MPs made of fiber had a smooth surface and a linear shape, while the MPs made of foam had rounder shapes. EDX spectra revealed that the primary component found in high concentration across all types of MPs was carbon, accounting for 83% of the weight on average, while oxygen accounted for 11% of the weight on average. In addition, Ca and Ti were found in fragments and fiber MPs, whereas the components of foam MPs were aluminum and chlorine.

In order to create an image, primary electrons are injected into a solid object, where they induce a variety of scattering processes (both elastic and inelastic), which are then collected by various detection systems (Bogner et al., 2007). When the specimen is passed through by back-scattered electrons, which increase in intensity as they pass through the specimen, information about the specimen’s topography and contrast (dependent on the material’s atomic number, Z) can be obtained. As the atomic number (Z) increases, the number of backscattered electrons increases proportionately. Sample compositions can be differentiated using this phenomenon, EDS which relates to SEM, which is an extra detector that provides qualitative and quantitative information on the elemental analysis of the sample.

This device uses an electron microscope cathode to generate an electron beam. Many interactions, including X-rays, are formed when the primary electron beam strikes the sample’s surface. The characteristics of the sample can be gleaned from the information on the elements and their spatial distribution as provided by the instruments. For understanding the accumulation, impact, and other factors affected by MP, Blair et al. (2019) used SEM analysis to evaluate the character and quantification of MPs. MPs from bank sediment samples at River Kelvin in Glasgow were size-fractionated: thus ranging from 2.8 mm to 11 m in diameter. The MPs were treated twice before they were subjected to extraction through density separation.

**FIGURE 4:**
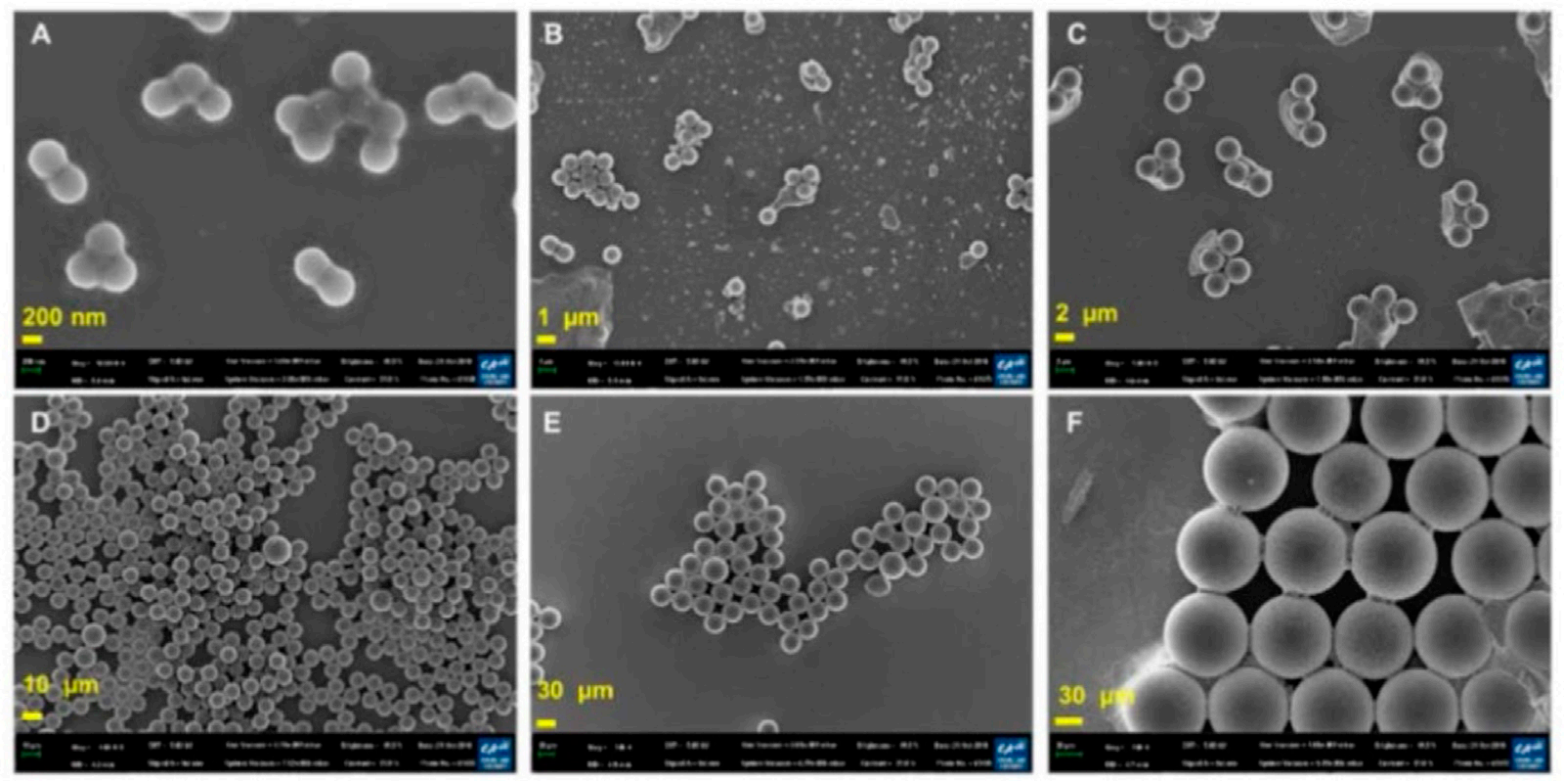
SEM images of PS particles. **(A)** 460 nm PS nanoparticles. **(B)** 1 μm PS particles. **(C)** 3 μm PS particles. **(D)** 10 μm PS particles. **(E)** 40 μm PS particles. **(F)** 100 μm PS particles (scale bar = 200 nm, 1, 2, 10, and 20 µm). Reprinted from Potential toxicity of polystyrene microplastic particles, Jangsun Hwang et al. (2020), with permission from Elsevier.

SEM is not only applicable to aquatic biota cells; it can be applied to blood cells in estimating MPs as reported by Chen et al. (2022). Meanwhile, a review by Hwang et al. (2020) also highlighted the use of SEM microscopic for morphological characterization. The morphologies of individual platinum-coated PS particles in aggregates were examined using SEM images (Figures 4A–F). Due to Van der Waals interactions with Na^+^ or Ca^2+^ in the buffer, smaller particles were more likely to assemble. While the zeta potentials of the other PS particles were closer to zero, the zeta potential of the 460 nm PS particles was −2.2 ± 0.1 mV (Figures 4E, F). The Derjaguin-Landau-Verwey-Overbeek (DLVO) theory might be able to explain the development of PS nanoparticle aggregates. The DLVO theory states that at a pH of 7, small particles carry less charge than large particles. Therefore, at a given ionic strength, the electrical double layer (EDL) repulsion forces between tiny particles are smaller.

However, SEM-EDS is widely considered a costly method compared to other microscopic analyses. The cost of SEM-EDS analysis may vary based on the complexity and size of the samples being analyzed, as well as the degree of significant effect required. Despite SEM-EDS can be a relatively expensive analytical technique, its high resolution and sensitivity make it a valuable instrument for a broad range of applications in materials science, geology, biology, and other disciplines. Indeed, for the characterization of MPs, it is sufficient to use SEM in ambient SEM mode, which avoids the use of gas-like nitrogen in the SEM chamber. Furthermore, despite being used to obtain high-resolution images, covering by sputtering gold or carbon on the sample surface can be avoided (Naji et al., 2019).

As an alternative to SEM, field emission scanning electron microscopy (FESEM) operates at a low voltage and allows for the acquisition of high-quality and high-resolution images of MPs materials without the need for special preparation prior to observation. There is no need to cover the sample with metal or carbon in this method, and the MP fragments are simply placed on the carbon tape on the aluminum stub. Nevertheless, the low voltage employed cannot be used to perform an EDS examination. When analyzing light elements, it is possible to use moderate energy for nonconductive particle analysis, avoiding the inclusion of additional signals in the energy dispersed from the X-ray spectroscopy (EDS) spectrum due to coverage. Nonetheless, additional energy is required, which may induce a charge effect in the sample. A review by Schmidt et al. (2019) revealed that SEM is a versatile device that can be used to describe MPs since it may relate to a STEM holder to analyze samples on a grid. Small particles can be observed without high energy and voltage as required by traditional transmission electron microscopy (TEM).

### Analytical method

#### FT-IR spectroscopy

Infrared spectroscopy, commonly known as IR spectroscopy, is a form of absorption spectroscopy that is frequently utilized in characterizing materials to investigate chemical bonding. When a molecule absorbs an infrared photon, it transits from the vibrational state (i.e., a characteristic of its ground state) to a vibrational state, which is typical of its excited state (Mariano et al., 2021).

**FIGURE 5:**
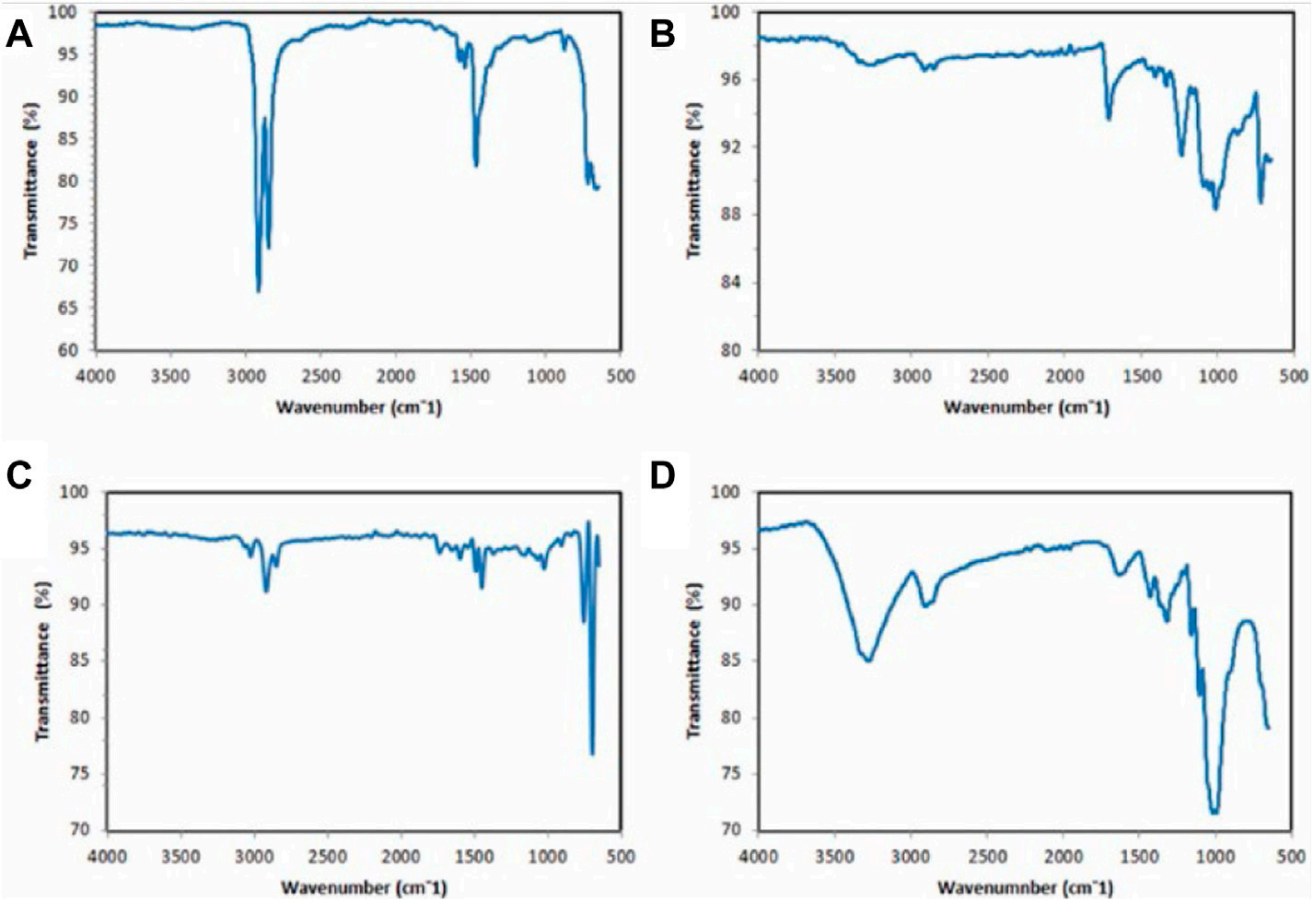
ATR-FTIR spectrums of polymers present in the types of MPs in Miri, Sarawak: **(A)** polyethylene (fragment) **(B)** polyester (fiber) **(C)** polystyrene (foam) **(D)** polypropylene (fragment). Reprinted from Occurrence, distribution and sources of microplastics in beach sediments of Miri coast, NW Borneo, Aliza Marai anak Alexander Tampang, Prasanna Mohan Viswanathan, (2022), with permission from Elsevier.

An interferometer is required to perform FTIR, also known as Fourier transform infrared spectroscopy. FTIR is frequently used to characterize MPs and the method can facilitate the scanning of all the frequencies included in the IR radiation originating from the source (Naji et al., 2019; Gopinath et al., 2020; anak Alexander Tampang and Mohan Viswanathan, 2022). In each investigation, MPs samples are excited, which results in the detection of vibrations that make it possible to obtain a spectrum with a fingerprint range as validated by anak Alexander Tampang and Mohan Viswanathan (2022) in Figure 5 below. This spectrum describes the nature of the material, and one may determine the material type by comparisons with established reference spectra. Larger particles greater than 500 nm can be analyzed using ATR-FTIR, but the use of micro-FTIR which allows for simultaneous viewing, mapping, and collection of spectra is required to analyze microscopic particles. The procedure can be performed in both the ATR mode and the reflectance mode (Mariano et al., 2021).

For thick samples, the reflectance mode is employed, although uneven particle surfaces can interfere with the analysis due to a refractive error (Harrison et al., 2012). Thus, only samples with specific properties can be evaluated; otherwise, subtractive mistakes prevent the observer from obtaining a valid signal. Furthermore, the lateral resolution is limited to a diffraction range, and samples larger than 20 m are not discernible (Löder et al., 2015). Figure 5 demonstrates how the IR spectra would change depending on the type of material being examined under the microscope. The method widely utilized by researchers for the identification of environmental pollutants involves focusing on the aromatic zone and searching for the C=C signal in the vinyl group. Meanwhile, styrene and polystyrene despite originating from the same functional group, usually represent different FTIR spectra after *in situ* sonochemical polymerization images as reported in a previous review (Hermán et al., 2015).

The average particle size of these polystyrene spheres is approximately 560 nm. There are multiple absorption peaks within the wavenumber range being considered. Since aromatic C-H stretching vibrations absorb energy, absorption peaks are observed at the wave numbers 3060.8 and 3026.0. Additionally, there are three absorption peaks at the wave numbers 1600.8, 1492.7, and 1452.2 because aromatic C=C stretching vibrations absorb energy (Hermán et al., 2015). These absorption peaks depict that benzene rings are present in the molecule. Absorption peaks at wave numbers 756.0 and 698.2 correspond to C-H out-of-plane bending vibration absorption, which indicates the presence of only one substituent in the benzene ring. This is indicated by the presence of only one absorption peak at each wave number (Hermán et al., 2015).

μ-FTIR has been frequently employed in MPs research to locate and describe MPs in sediment (Blair et al., 2019; Ding et al., 2019; Jiang et al., 2019; Gopinath et al., 2020; anak Alexander Tampang and Mohan Viswanathan, 2022), marine creatures (Prata et al., 2019; Dai et al., 2022), and surface water (Wagner et al., 2014; Fan et al., 2019; Zhou et al., 2020). The approach collects IR signals with great spatial resolution and is suitable for the characterization of complicated samples. For example, Ding et al. (2019) conducted a -FTIR analysis on MP pollution in surface sediments in Wei River, and identified eight polymer types such as rayon, PS, PP, PA, PET, PE, PMMA, and PU. The researcher provided a definitive validation of FTIR to identify ingested plastic polymer types in water samples and sediments. Most of the MPs found in the tWei River were of very small size (less than 0.5 mm), accounting for 68.1% of the total. This result reflects the vitality of the Fourier transforms infrared spectrometer for the determination of PS and other polymers.

#### Raman spectroscopy

Jiang et al. (2019) found that Tibet Plateau used micro-Raman spectroscopy to identify five distinct forms of MPs, each of which had a distinct chemical composition. A significant proportion of microscopic MPs were found to be fibrous and clear. PS and other MPs such as polyethylene terephthalate (PET), polyethylene (PE), polypropylene (PP), and polyamide (PA) were found (Jiang et al., 2019).

**FIGURE 6:**
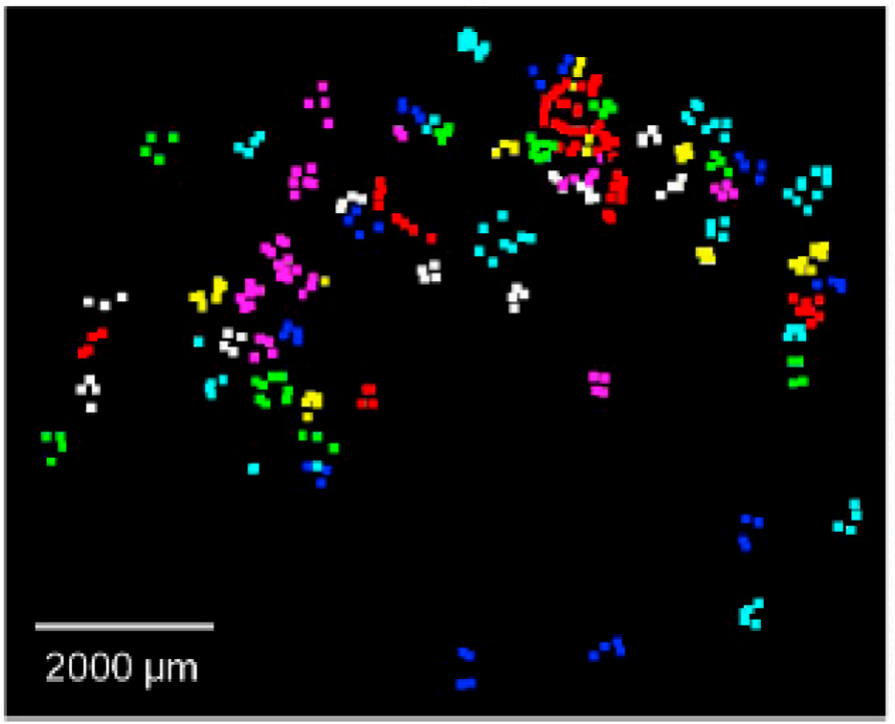
Retrieved microplastics were counted and identified to be PP (purple), PE (cyan), PS (yellow), PA (white), PMMA (green), PET (red) and PVC (blue). Reprinted from Improved Raman spectroscopy-based approach to assess microplastics in seafood, Matthew Ming-Lok Leung et al. (2021), with permission from Elsevier.

The Raman spectroscopy method is a technique that is based on the interaction of radiation and the substance being studied. This method makes use of laser radiation that interacts with the vibrational motions of the molecules and produces the re-emission of light at wavelengths that are characteristic of that particular atomic group (Leung et al., 2021; Nava et al., 2021). This is accomplished by using the property of molecules. Based on this approach, a Raman spectrum is produced by the inelastic scattering of photons by the molecules that make up the sample, which occurs when incident light interacts with the sample as shown in Figure 6.

When a monochromatic laser beam at frequency ^υ^_0_ is placed on the sample, a portion of the radiation is elastically diffused at the same beginning frequency υ_0_, which means that it contains photons with the same energy (a phenomenon defined as Rayleigh scattering). As a result of inelastic diffusion, the spectrum of the dispersed radiation will also contain several lines with a frequency that is either higher or lower than that of the Rayleigh line (Nava et al., 2021).

**FIGURE 7:**
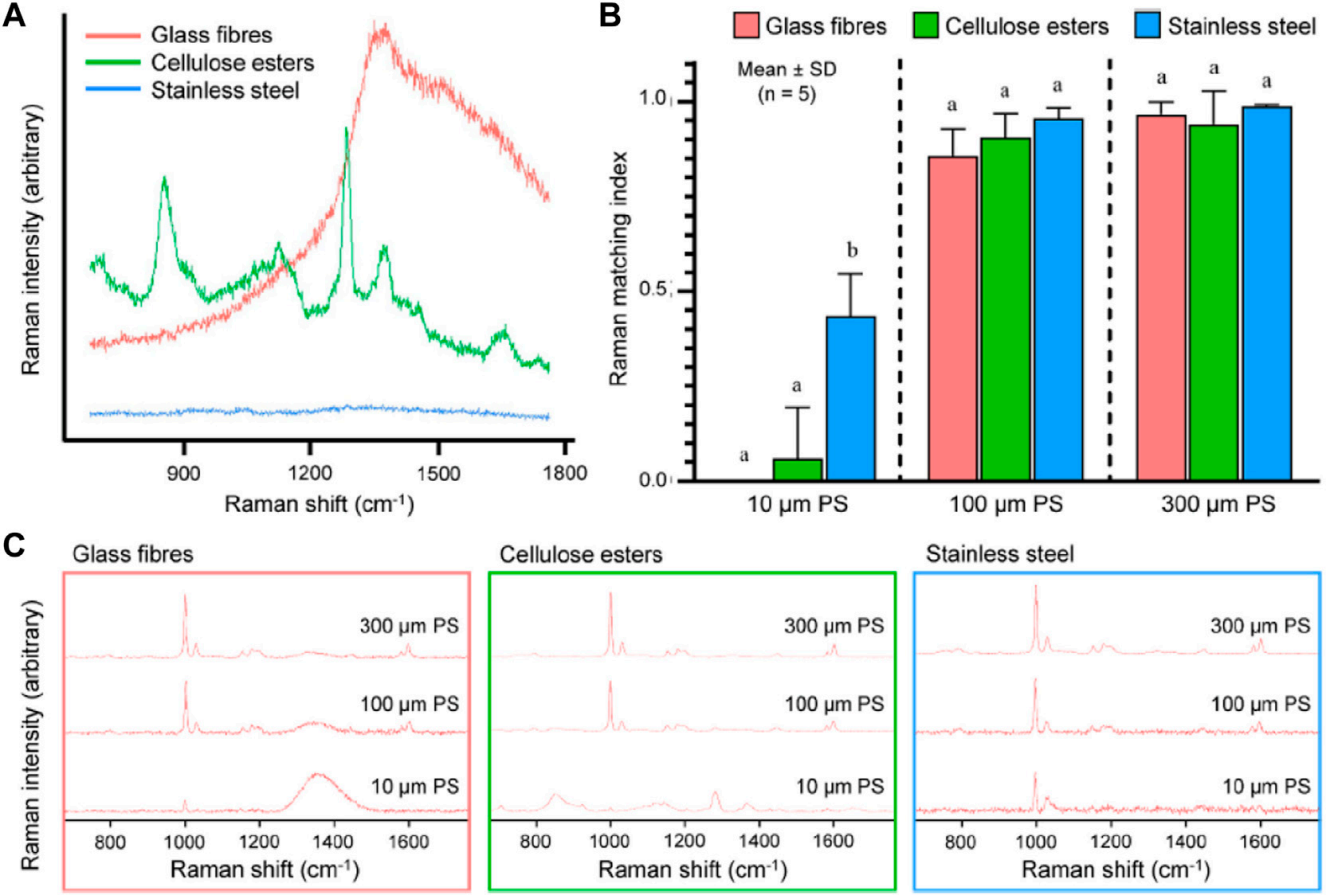
**(A)** Raman spectra of filter membranes made of glass fibers, cellulose esters and stainless steel excited at 785 nm; **(B)** Raman matching index of polystyrene (PS), determined for three particle sizes (10, 100, and 300 μm) and placed on the three types of filter membranes presented in **(A)**; **(C)** Raman spectra of PS of the three particle sizes placed on the three materials excited at 785 nm using a ×10 objective (NA = 0.25). Reprinted from Improved Raman spectroscopy-based approach to assess microplastics in seafood, Matthew Ming-Lok Leung et al. (2021), with permission from Elsevier.

A comprehensive review by Leung et al. (2021) aimed to identify the suitable filter substrate for Raman spectroscopy (Figure 7). It was found that the membranes that were built of cellulose esters revealed large peaks at approximately 900, 1,300, and 1,400 cm^−1^, but the membranes composed of glass fiber displayed a broadband that was centered around 1,400 cm^−1^. However, it appeared that the membranes made of stainless steel were not sensitive to the Raman excitation given the lack of detectable peaks (Figure 7A). The interference caused by employing these filter membranes as the substrate materials in Raman spectroscopy in the process of identifying microplastics was evaluated with PS consisting of three different particle sizes as illustrated in Figures 7B, C. The various types of filter membranes and PS particle sizes were compared using the matching index, which indicated how effectively the polymer was identified.

Raman spectroscopy is advantageous over FTIR spectroscopy. It provides a non-destructive study of materials in any aggregate with less complex sample preparation, utilizing lasers of various power to influence the substance, and yielding a Raman spectrum characteristic of the examined material. Furthermore, the thickness of the samples has no bearing on the result. Raman spectroscopy can be employed to analyze samples in solution, gas, film, surface, solids, and single crystals, as well as at various temperatures. The low-temperature spectra (10K) allow: 1) to limit any sample damage caused by local heating induced by the laser; and 2) comparisons with studies acquired using other technologies that produce satisfactory findings at low temperatures. Among the drawbacks, fluorescence can be a major issue when combined with Raman spectroscopy. The photon that is emitted in both the Raman and the fluorescence phenomena originates from excitation in the absorption band. Furthermore, the quantum yield of the fluorescence is frequently higher by an order of magnitude than the intensity of the Raman diffusion. The Raman and the fluorescence are closely related, however, some of the interference caused by fluorescence can be eliminated by using a different algorithm or more effective detectors (Ribeiro-Claro, Nolasco, and Araújo, 2017). Table 1 below summarizes the studies involving contamination by polystyrene and its concentrations and sources.

**TABLE 1:** Summary of polystyrene studies in freshwater ecosystems across the world.

Area of study	Sample source	Abundance of MP (concentrations)	Route/Source of polystyrene into aquatic environment	Method identification of polystyrene	Citation
North of Chennai city, India	Water and sediment	5.9 particles/L (water) and 27 particles/kg (sediment)	Water treated does not undergo micro plastic removal process	Fourier Transform Infrared Spectroscopy (FTIR)	Gopinath et al. (2020)
Wei River, China	Wei River’s surface water and sediments	3.67 to 10.7 items/L (surface waters) and 360 to 1,320 items/kg (sediments)	Unknown	FTIR	Ding et al. (2019)
Tibet Plateau	Surface water and sediment from 6 sampling sites along five different rivers	483 to 967 items/m^3^ (surface water) and 50 to 195 items/kg (the sediment)	Concentrated in both remote and highly developed area	μ- Raman Spectroscopy	Jiang et al. (2019)
River Kelvin in Glasgow, West of Scotland	Sediment samples from the River	161–432 MPs kg^−1^dry sediment	Drivers of river fine sediment dynamics	Scanning electron morphological analysis (SEM)	Blair et al. (2019)
Miri Coast, Sarawak Malaysia	Sediment samples from eight different beaches	1,305 particles/90g	Food packaging and boxes	Analyzed through stereoscopic microscope, ATR-FTIR and SEM-EDX	anak Alexander Tampang and Mohan Viswanathan (2022)

## Toxicological impact of polystyrene on the aquatic environment

### Factor influencing presence of impact from polystyrene

The effects of PS have been proven to rely not only on their sizes and forms although some fibers have exhibited stronger deleterious effects than beads (Ziajahromi et al., 2017), but also on their concentrations and time of exposure. The size of the MP particles is the primary component that determines the hazardous effect of MPs on microalgae. Particles with a size of 0.1 μm or less are thought to cause primarily chemically-mediated effects, whereas particles with a size of 2 μm or more are believed to cause primarily physically-mediated effects.

When treated with *Chlorella pyrenoidosa*, Yi et al. (2019) discovered that the cytotoxicity of PS particles depended on their size (the IC50 for 0.55 m PS was 9.10 mg/L) but no toxicity was observed for 5.0 m PS. These findings are consistent with previous reports in which the toxicity of PS increased with particle size (Parsai et al., 2022). This result demonstrates that the shading effect is not the primary reason MPs are hazardous to microalgae.

Aside from differences in the inhibition of growth rate, there are also discrepancies in the effect on the parameters of photosynthesis. For example, Sjollema et al. (2016) discovered that the photosynthetic characteristics of *Dunaliella tertiolecta* did not significantly vary when the microalga was exposed to three different sizes of PS beads (0.05, 0.5, and 6 m). The PS beads ranged in size from 0.05 to 0.5 μm Bhattacharya et al. (2010) earlier suggested that the shadowing effect generated by bigger size MP is the primary reason for the suppression of photosynthesis, but the evidence suggests otherwise.

The most recent findings provide more insights into this contentious debate. For example, Liu G et al. (2020) investigated the effects of five different types of PS with different sizes (0.1, 0.5, 1, and 2 m), and discovered that large-size MPs significantly decreased the light conversion efficiency in the photochemical reaction center and the energy conservation parameter on *Scenedesmus obliquus*. The scientists concluded that the sizes of the MPs have a substantial impact on the photosynthesis of microalgae, with large sizes of MPs resulting in reducing the amount of light converted to chemical energy in the photochemical reaction center due to light blockage (Liu G et al., 2020).

Subsequently, several forms of MPs with varying inhibitory rates have been documented. The effect of different MPs (PS, PE, and PVC) of the same size (74 m) on the growth of *Skeletonema costatum* was investigated by Zhu et al. (2019). The researchers demonstrated that the toxicity of these MPs was associated with their chemical category, specifically their hydrophobicity. Given their hydrophobic properties, some MPs precipitated from water reduced their harmful effects on microalgae cells (Zhu et al., 2019).

A particle’s toxicity might also vary based on its surface charge (negative or positive). Sjollema et al. (2016) employed carboxylated PS (PS–COOH) microbeads (0.5 m) to assess type-dependent effects on three microalgae (*Dunaliella tertiolecta*, *Chlorella vulgaris*, and *Thalassiosira pseudonana*). Negatively-charged beads had no to negligible (10%) effects on photosynthetic efficiency. Meanwhile, 50 nm cationic amino-modified PS (PS-NH_2_) generated nano-scale aggregates (Z-Average 200 nm), inhibiting *D. tertiolecta* development (EC50 = 12.97 g/mL) (Liu Y et al., 2020). Casado et al. (2013) discovered that 55 and 100 nm positive surface-charged PS inhibited the growth of *Pseudokirchneriella subcapitata* (EC50 of 0.58 and 0.54 g/mL, respectively). These observations depicted that surface amino groups affect the toxicity of polymers (Casado et al., 2013; Bergami et al., 2017). Positively-charged particles absorbed by endocytosis alter the plasma membrane by creating holes (“proton sponge” idea).

Wang et al. (2017) discovered that aged PS and PVC have greater inhibition than virgin (29.10% and 16.72%). The authors described this toxicological increase of aged MPs because of surface alterations, including high porosity resulting in surface oxidation, minor partial micro-cracks, and higher crystallinity, which encourage the breakdown into smaller pieces, and bigger specific surface area. When MPs and components are leached, they can interact with aquatic biota of all trophic levels, such as microalgae (Parsai et al., 2022) and aquatic animals like fish, tadpoles, and *Eriocheir Sinensis* (crab) (Rowley et al., 2020). The quantity of polystyrene particles, their bioavailability to planktonic, nektonic, and bottom-dwelling freshwater biotas, as well as their extensive relative abundance in freshwater compartments, can unavoidably lead to ingestion by different biological creatures (Law et al., 2010). These organisms include diatoms, planktonic crustaceans, fish, mussels, and zooplankton.

Liu et al. (2019) demonstrated that all PS particles suppressed the development of *Scenedesmus obliquus* upon short-term exposure with doses, but the effect of small-sized MPs (such as PS 0.1 m and PS-NH_2_ 0.1 m) was not persistent. The microalgae recovered after 199 h of exposure to small-sized PS. Zhu et al. (2019) reported that MPs decreased the photosynthetic ability of *Skeletonema costatum* over time. Meanwhile, in terms of the concentrations of polystyrene; Chae et al. (2019) evaluated the effects of high concentrations of large PS particles (approximately 200 m in diameter) on *Dunaliella salina* and reported its growth-promoting and photosynthetic activity.

Overall, the mode of action of MPs against algae seems to be mostly size-dependent. Large-sized plastics inhibit photosynthesis in algae by causing light obstruction, but small-sized plastics, such as nano plastics adsorb through the cell wall and cause chemical reactions, which alter the growth rate and cell function. This pattern depends on MP concentration, type, exposure time, and microalgae species.

### Noxious impact of polystyrene on fishes

With all the factors being considered, the toxicity impact of polystyrene on the aquatic environment organism has been proven with research evidence. Li et al. (2021) discovered that fish fed by swallowing took in more pellets than those fed by filtering or sucking. All related studies using high-definition and high-speed observational techniques found that all species passively sucked in microfibers during breathing, rather than actively capturing microfibers. While the number of ingested microfibers increased in the food presentation, some of the microfibers remained in fish gastrointestinal tracts and gills. The present findings suggest that fish eat MPs accidentally rather than on purpose, which is a common occurrence (Li et al., 2021).

A study found that polystyrene altered the fish’s transcriptional gene and immunological responses, as well as their behavior (*Danio rerio*) (Limonta et al., 2019). Experiments on zebrafish exposed to two doses of HDPE and PS MPs for 20 days reported that the fish were affected. Transcriptomic data revealed a link between changes in immune system gene expression and gene downregulation, including changes in epithelial integrity and lipid metabolism. Pathogens from PS and HDPE enter epithelial barriers, which eventually increases the risk of infection at the mucosal locations. Gills and intestinal epithelium demonstrated similar modifications in tissue composition and an increase in neutrophil numbers, secondary lamellae adhesion and partial fusion, and mucous hypersecretion. Notably, another study using zebrafish found that exposure to 1,000 g/L of PS MP led to increased mucous secretion in the GIT, which was then followed by an increase in the number of pathogenic bacteria in the gut microbiota. Pathogen-inducing effects of MPs on the gut microbiota could explain the transcriptional regulation of immune responses seen in the current investigation of MPs (Liu et al., 2019). An organism’s resistance to infections could be weakened if the impacts on mucosal epithelial integrity and immune response are compromised, as suggested by transcriptomic and histological detection. This proves that the effect of PS on zebrafish’s biological features is undeniable. In the study conducted by Benson et al. (2022), the organisms *Daphnia magna* and *Pimephales promelas* were subjected to polystyrene microplastics for 5 days. Both the bioconcentration factor and the bioaccumulation factor were quite low for both species. Polystyrene that was polluted with nickel (Ni) caused abnormalities in *Daphnia Magna* (Kim) such as immobilization and morphological changes in the organism. In another study, polystyrene was confirmed to reduce fish activity, measured by the distance traveled and area covered, and triggered histological abnormalities in fish livers. These nano plastics also pierced the embryo walls and hatched juveniles’ yolk sacs (Chae et al., 2018).

In another study, a combination of food and plastic particles of varying sizes was used to nourish specimens of the genus *Dugesia japonica*. Although the enterocytes were found to be unaffected in terms of their capacity to regenerate, at least a portion of the microplastic was phagocytized by the cells. Under chronic exposure, a considerable reduction in the thickness of the gut epithelium and the lipid content of enterocytes, as well as the induction of apoptotic cell death, and a slower growth rate, were detected. Since microplastics are very adaptable to both chemical and mechanical stress, they were able to disrupt the homeostasis of the planarians. This can be explained by the fact that microplastics are very flexible (Gambino et al., 2020). According to Zwollo et al. (2021), rainbow trout (*Oncorhynchus mykiss*) when exposed to polystyrene microplastics recorded damaged growing B cells in basic cultures of the anterior kidney growing immune, lowered gene expression RAG1, and altered the membrane shape of immunoglobulin heavy chains μ and tau. Chronic exposure to PS microplastics may cause vertebrate species to elicit inadequate IgM/IgT responses to infections. Humans and other higher vertebrate species are likely to be affected by PS microplastics because of the largely conserved B-lymphopoiesis of vertebrates.

In another study conducted by Assas et al. (2020), an alteration in the expression of genes involved in cell adhesion was observed once medaka fish were subjected to polystyrene MP. Specifically, xenobiotic metabolic processes, brain development, and other activities in the intestines were detected. However, the survival rate and reproductive rate of *Japanese medaka* were not significantly affected in any way by the exposure. These findings reflect the potential of virgin PS-MPs to accumulate in the intestines of medaka, but their toxicity to the fish is rather low at concentrations of up to 10 beads per liter.

### Noxious impact of polystyrene on microalgae

Microalgae are fundamental to marine ecology and play a crucial role in species equilibrium. Microalgae also have substantial commercial significance. Due to their function as primary producers, they have a place of utmost significance among the diverse range of marine creatures that are susceptible to the adverse effects of MP (Jiang et al., 2019; Ning et al., 2022). Microalgae, which can range in size from a few micrometers (m) to a few hundred micrometers, have a significant potential for interacting with MP in aquatic environments due to their size (Liu et al., 2019).

MPs have been shown to reduce chlorophyll content and photosynthetic activity (Bhattacharya et al., 2010; Mao et al., 2018), which is self-reliant on growth inhibition (Besseling et al., 2014) and shading effect (Bhattacharya et al., 2010). Furthermore, MPs may impair photosynthesis by altering the electron donor site, the center of photosynthesis reaction, which is accountable for energy conversion and the electron carrier chains. This can lead to the buildup of electrons and the generation of reactive oxygen species (ROS), which causes oxidative stress (Bhattacharya et al., 2010; Mao et al., 2018).

Additionally, MPs may cause direct physical damage, depletion of nutrients, an increase in osmotic pressure, and the release of toxic chemicals (Besseling et al., 2014; Liu et al., 2021). These effects have been documented by several research groups. In addition, microplastics have the potential to cause morphological changes in microalgae such as an unclear pyrenoid (non-membrane bound organelles found in chloroplasts of algae and hornwort plants) and the detachment of plasma from the cell wall, deformed thylakoids, and cell wall thickening (Mao et al., 2018), which are to be internalized during the cell division (Chae et al., 2019) or consumed by mixotrophic organisms (Long et al., 2015). Nevertheless, all these effects seem to be only temporary as they are accompanied by adaptive responses leading to recovery. These responses include membrane thickening, homo-aggregation (to limit surface exposure), and hetero-aggregation.

This section focuses only on the impact of polystyrene on microalgae. According to Xiao et al. (2020), the physiological and transcriptional responses of freshwater microalga *Euglena gracilis* were altered by polystyrene. Polystyrene microplastics were discovered to cause superoxide dismutase and oxidative stress, thereby reducing the amount of pigment in the body and altering gene expression patterns at the molecular level. These genes are involved in cellular processes, processing genetic information, body systems, and metabolisms. The severe effects might be caused by the KCS and CTR1 genes. Gushchina et al. (2020) found that common freshwater algae had less than two essential fatty acids, specifically linoleic and linolenic acids. In another study, Liao et al. (2020) identified the morphology of *E. gracilis* when exposed to small and big PS microbead sizes. Surprisingly, small-size PS microbeads had the same repressive effects on *E. gracilis* as large-size PS microbeads. The authors noted that the morphology *of E. gracilis* cells (ellipsoidal or spheroidal) and their larger size (32–55 m in length and 8–9.6 m in diameter) may affect the results (Guschina et al., 2020; Xiao et al., 2020; Parsai et al., 2022).

Meanwhile, the adsorption of polystyrene particles onto a model cellulose film and two living algae species, *Chlorella* and *Scenedesmus*, has been investigated. This adsorption was observed to favor positively-charged PS beads over negatively-charged PS beads due to electrostatic attraction between the microbeads and the cellulose constituent of the model and living systems. Such a charge preference is particularly obvious for *Chlorella* and *Scenedesmus*, in which their contact with plastic beads was also dependent on algal morphology and motility as measured by Freundlich coefficients (Bhattacharya et al., 2010). PS beads affect the adsorption, photosynthesis, and ROS production effect, and these effects vary with the charge. Further impacts are presented in detail in Table 2.

**TABLE 2:** Effect of polystyrene exposure to fishes and microalgae based on several effect criteria.

Scientific name	Common name	Summarized impact	Citation
*Danio rerio*	Zebrafish	Transcriptional gene	Lei et al. (2018) and Liu et al. (2019)
		Immunological responses	
		Character like gills adhesion and increasing of neutrophil numbers	
*Daphnia Magna*	Planktonic crustacean	Low bio concentration and bioaccumulation factor	Chae et al. (2018) and Benson et al. (2022)
		Changes in morphology	
		Fish activity decreased by distance and area caused by the alteration of fish liver histology	
		Pierced embryo walls and hatched juveniles’ yolk sacs	
*Pimephales promelas*	Fathead minnow fish	Low bio concentration and bioaccumulation factor	Benson et al. (2022)
*Dugesia japonica*	Species of dugesiid triclad	Major change in the thickness of the gut epithelium	Gambino et al. (2020)
		The amount of fat in the enterocytes	
		Slower growth rate	
*Oncorhynchus mykiss*	Rainbow trout	Damaged growing B cells	Zwollo et al. (2021)
		Lowered gene expression RAG1	
		Changed the membrane shape of immunoglobulin heavy chains μ and tau	
		Inadequate IgM/IgT responses to infections	
*Oryzias latipes*	Medaka fish	Alteration of xenobiotic metabolic process, brain development, and other activities in the intestines	Assas et al. (2020)
		The survival rate and reproductive rate remained unchanged	
*Euglena gracilis* ^a^	Single-celled alga	Affected its physiological and transcriptional responses	Guschina et al. (2020), Xiao et al. (2020), and Parsai et al. (2022)
		Lessened the amount of pigment in the body	
		Changed the way genes are expressed in molecular level	
		Less of two essential fatty acid; linoleic and linolinic	
		Caused superoxide dismutase and oxidative stress	
*Chlorella* sp.^a^	Freshwater species of green microalga	PS positively charged (PS^+^)	Bhattacharya et al. (2010)
		Higher in adsorption and ROS production	
		PS negatively charged (PS^−^)	
		Significant decrease in photosynthesis when concentration of polystyrene beads higher than 1.8 mgL^−1^	
*Scenedesmus* sp.^a^	Freshwater species of green microalga	PS positively charged (PS^+^)	Bhattacharya et al. (2010)
		Higher in adsorption and ROS production	
		PS negatively charged (PS^−^)	
		Decrease up to 40% in photosynthesis	
*Scenedesmus obliquus* ^a^	Freshwater green algae species of the genus Scenedesmus	Having growth inhibition by 2.5% at 1,000 mgL^−1^	Besseling et al. (2014)
		Chlorophyll content decrease when concentration higher than 100 mgL^−1^	
*Dunaliella tertiolecta* ^a^	Saltwater small colorless refractive globules throughout the cytoplasm	PS positively charged (PS^+^)	Bergami et al. (2017)
		Having growth inhibition by when EC_50_ at 12.97 mgL^−1^	
		Having aggregation	
		PS negatively charged (PS^−^)	
		Having growth inhibition up to 25.4%	
		Having aggregation	
*Chlamydomonas reinhardtii* ^a^	Freshwater single-cell green alga	PS negatively charged (PS^−^)	Chae et al. (2018)
		No effect on growth inhibition	
		Having internalization in cell division	
*Amphora* sp.^a^	Freshwater major genus of marine and diatoms	Having aggregation as induce assembly	Chen et al. (2011)
*Tisochrysis lutea* ^a^	Saltwater species in to feed oyster and shrimp larvae	Growth inhibition, chlorophyll, and heteroaggregation unaffected	Long et al. (2015)
*Chaetoceros neogracile* ^a^	Saltwater phylogenetically distinct from an Antarctic species	Having growth inhibition, chlorophyll content and heteroaggregation effect	Long et al. (2015)
*Rhodomonas baltica* ^a^	Saltwater genus of cryptomonads	Significant inhibition of cell count and significant decrease on chlorophyll content	Lyakurwa (2017)
*Chlorella pyrenoidosa* ^a^	Freshwater green alga	Having growth inhibition until day 22 till recovery	Mao et al. (2018)
		Having photosynthesis inhibition until day 6–8 of recovery	
		Damaged membrane, unclear pyrenoid	
		Distorted thylakoid, wall thickening at day 13, but managed to recover	
*Dunaliella tertiolecta* ^a^	Saltwater	PS neutral charged (PS^0^)	Sjollema et al. (2016)
		Having growth inhibition up to 57% at 250 mgL^−1^	
		Photosynthesis unaffected	
*Chlorella vulgaris* ^a^	Freshwater	PS negatively charged (PS^−^)	Sjollema et al. (2016)
		No effect on photosynthesis	
*Microcystis aeruginosa* ^a^	Freshwater cyanobacteria which can form harmful algal blooms of economic and ecological importance	Inconsistent increasing of cell count	Yokota et al. (2017)
		Inconsistent decreasing of algae size	
		Growth inhibition, growth biomass and growth colonization unaffected	
*Dolichospermum flos-aquae* ^a^	Species of freshwater cyanobacteria belonging to the family *Aphanizomenonaceae*	Inconsistent increasing of cell count	Yokota et al. (2017)
		Inconsistent decreasing of algae size	
		Decreasing of filament	
		No effect on elongation	
		No effect on growth inhibition	
		Having effect on biomass and colonization	

Polymer: PS, polystyrene: when available, particle charge is presented as (+) positive, (0) neutral or (−) negative.

^a^ microalgae

*Scenedesmus obliquus*, which is species of the genus *Scenedesmus* had a different impact when exposed to natural PS. *Scenedesmus obliquus* with PS particles displayed lower population expansion by 2.5% at 1,000 mgL^−1^ and lower chlorophyll concentrations. Such plastic concentrations are significantly greater relative to what is currently reported for marine and freshwater. Nevertheless, their presence in sediment pore fluids may be confirmed in the future (Besseling et al., 2014). A study by Bergami et al. (2017) proved that the green prey microalga *D. tertiolecta*, which was the subject of a study was affected by the presence of 40 nm PS anionic carboxylated (PS-COOH) and 50 nm cationic amino-modified (PS-NH) NPs on planktonic species. They were tested for long-term toxicity to microalgae over 14 days and 72 h. PS-COOH generated micro-scale aggregates (Z-Average>1 m) and did not affect the growth of microalgae up to 50 g/mL. On the other hand, PS-NH_2_ generated nano-scale aggregates (Z-Average 200 nm), which inhibited algal growth (EC = 12.97 g/mL).

**FIGURE 8:**
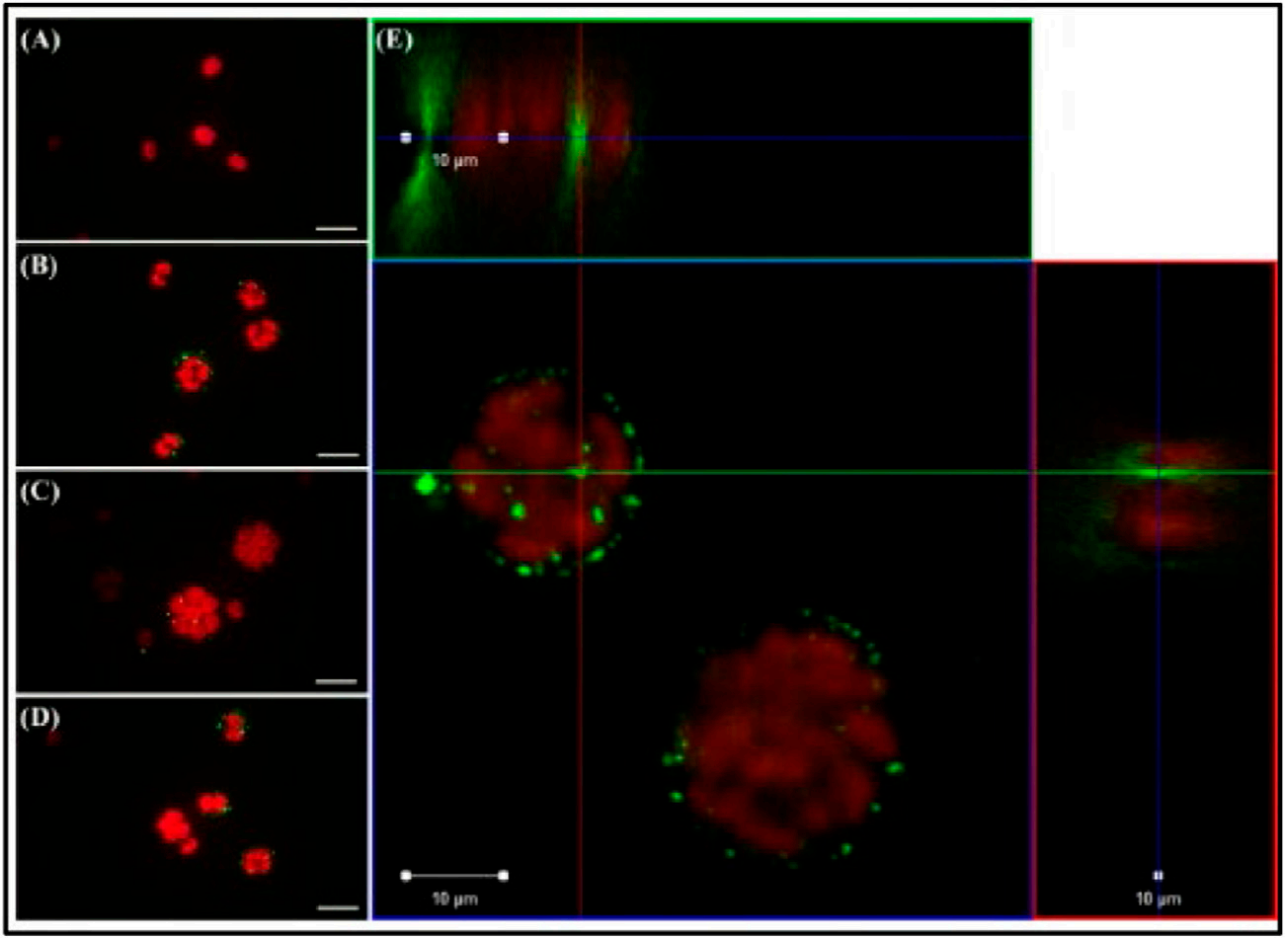
Observation via optical microscopy **(A–D)** and confocal laser scanning microscopy **(E)** of the alga *Chlamydomonas reinhardtii* (red emissions) directly exposed to nano-sized polystyrene (NPS; green emissions) for 72 h. Scale bar = 20 **(A–D)** and 10 μm **(E)**. Reprinted from Trophic transfer and individual impact of nano-sized polystyrene in a four-species freshwater food chain, Yooeun Chae et al. (2018), with permission from Elsevier.

The single-cell green alga *Chlamydomonas reinhardtii* was the subject of a study by Chae et al. (2018), focusing on the effects of nano-sized polystyrene plastics (NP-PS), which were constantly exposed to a freshwater ecosystem to study trophic transmission, influence, and embryonic uptake (Figure 8). In the trophic transfer test, algae were fed a diet that contained 50 mg/L nano plastics (plastic particles 100 nm in size). Each species was exposed directly to nano plastics. Nanoplastics have been reported as the main producer in the digestive organs of species higher up the food chain. Because *C. reinhardtii* had been exposed to NP-PS, the plastic particles were stuck to its surface. Unlike the control group (Figure 8A), the exposed groups (Figures 8B–D) had separated red (algae autofluorescence) and green (NP-PS fluorescence) emissions. Chae et al. used confocal laser scanning microscopy (CLSM) to confirm that NP-PS permeated the outer layer of *C. reinhardtii* during cell division and stuck to the surface of zoospores (Figure 8E).

NP-PS had no effect or caused any growth inhibition; however, it affected cell division by internalization. In a study conducted by Chen et al. (2011) on the influence of ENs on exopolymer substances (EPS) microgel formation, different quantities of ENs (polystyrene nanoparticles) were added by the researchers with 0, 10, and 100 ppb concentrations. ENs can effectively stimulate microgel assembly in Amphora, resulting in microgels with an equilibrium size of 4–6 m and low protein-to-carbohydrate ratios. A low protein-to-carbohydrate ratio indicates the absence of detectable protein and deficiency of hydrophobic regions. Notably, 100 ppb ENs were required to initiate considerable EPS construction in EPS with a low protein (hydrophobic) percentage, such as *Amphora* sp. This event causes hetero-aggregation and assembly. This result depicts the specific features of polystyrene-based engineered nanoparticles (ENs), which contribute to their alluring commercial uses and raises concerns about environmental safety. Long et al. (2015) evaluated two algae species, *Tisochrysis lutea,* and *Chaetoceros neogracile,* and revealed that the former did not affect growth inhibition, chlorophyll content, or the hetero-aggregation of the organism. Meanwhile, the result demonstrated a different response to *Chaetoceros neogracile* characterized by signs of growth inhibition, varying levels of chlorophyll content, and hetero-aggregation. The interaction of algae species and various MP beads has also been investigated (Lyakurwa, 2017) in terms of the uptake of 10 m virgin polystyrene (PS) microbeads and 1–5 m of glucose-responsive fluorescent (GF) by the *dinoflagellate Oxyrrhis marina Dujardin* (12–30 m). The researchers also assessed the effects of 10 m virgin polystyrene (PS) microbeads on cryptophyte *Rhodomonas baltica Karsten*. *Rhodomonas baltica Karsten*, a saltwater alga, reflected a significant decline in the number of cells and the amount of chlorophyll, which is interesting to investigate.

Another freshwater green alga, *Chlorella pyrenoidosa,* was examined to evaluate its growth inhibition using microplastic diameters of 0.1 and 1.0 m and three concentration gradients that covered (10 and 50 mg/L) and exceeded (100 mg/L) its ambient concentrations, respectively. PS microplastics inhibited *Chlorella pyrenoidosa* growth from the lag to the earlier logarithmic stages, but there was a modest difference in the peak inhibition ratio (about 38%) between the two microplastic sizes. In addition to lower photosynthetic activity in *Chlorella pyrenoidosa*, researchers discovered unclear pyrenoids, twisted thylakoids, and a damaged cell membrane, all of which were attributed to physical damage and oxidative stress produced by microplastics. Surprisingly, *Chlorella pyrenoidosa* may mitigate the negative impacts of microplastics by cell wall thickening, algae homo-aggregation, and algae-MP hetero-aggregation, increasing algal photosynthetic activity, growth, and normal cell structures (Mao et al., 2018).

Sjollema et al. (2016) investigated the effects of polystyrene on *Chlorella vulgaris* and *Dunaliella tertiolecta* after exposure to specific contaminant beads*. C. vulgaris* is a type of algae that lives in freshwater, while *D. tertiolecta* is an example of an alga that lives in saltwater. Despite both being exposed to negatively charged polystyrene, none of the factors influenced the photosynthesis of the other. However, when confronted with PS that was either neutral or negatively charged, the growth of algae in saltwater was inhibited whereas algae in freshwater did not display the same response.

Yokota et al. (2017) studied both types of freshwater algae, namely *Microcystis aeruginosa* and *Dolichospermum flosaquae*. When exposed to polystyrene, both organisms depict an inconsistent rise in cell count and decrease in the size of the algae, but neither exhibited any growth-suppressing effect. The additional influence of *Dolichospermum flosaquae* is by shortening the filament; nonetheless, this alteration did not affect the elongation. *Dolichospermum flosaquae*, on the other hand, exhibited an increase in both their biomass and colonization in contrast to *Microcystis aeruginosa*. Additionally, extensive research is required to elucidate the effects on other aquatic creatures for a broader scope. Most toxicology investigations employ overdosed exposure settings and disregard the fact that data on the translocation of plastics into internal organs sometimes rely on artifacts.

## Conclusion and future perspective

MPs and NPs are found everywhere in the environment, and the cumulative intake of these polymers by humans will continue to increase in the years to come. The abundance of MP in the aquatic environment worldwide has been covered in this review to provide substantial evidence of the accumulation of MPs and their exposure to the public. Apart from humans, exposure to MPs has triggered fears of long-term acute toxicity to fish and algae. Based on the comparison of previous studies, this review concluded that most of the ingestion routes of MPs occur via the freshwater ecosystem. These MPs find their way into freshwater and are unable to eliminate by water treatment, thus highlighting the need for further investigation of effective wastewater treatment plants. The methods evaluating the presence of PS were presented in this review, which was broadly categorized into microscopy and analytical methods. Meanwhile, the types of microscopy methods include stereoscopy and SEM morphology while analytical methods such as FT-IR and Raman spectroscopy can be employed for characterization.

By focusing on only polystyrene, its severity on aquatic behavior was tabulated and summarized for further understanding of the implications of noxious polystyrene. Some of the impacts on fishes encompass immunological responses, low bio-concentration and bioaccumulation factors, morphological changes, and slow growth rate. Meanwhile, the impact on algae includes reducing the amount of pigment in the body, higher adsorption and production of ROS, a decrease in photosynthesis with chlorophyll content, and inducing an aggregation effect. In conclusion, there is a significant need for additional research on the mechanisms operating at the cellular and tissue levels of algae, as well as the long-term implications in tissue and organ accumulation on fishes.

## Knowledge gap

Research on marine microplastics is further along than research on freshwater microplastics, which has large information gaps. There is a lack of complete data on their abundance for major surface waters, and there is no data at all for tiny surface waterways. In a similar manner, additional research is required to determine the roots of the problem and its impact on the ecosystem. There are no data on the biological effects that MP has on the creatures that live in freshwater. Since eating can result in increased chemical exposure, the deposition of additional freshwater contaminants on MP is a source of significant worry. Once more, there are no facts available on this extremely important subject.
